# Caregiver-mediated exercises with e-health support for early supported discharge after stroke (CARE4STROKE): study protocol for a randomized controlled trial

**DOI:** 10.1186/s12883-015-0440-z

**Published:** 2015-10-09

**Authors:** Judith Vloothuis, Marijn Mulder, Rinske H M Nijland, Manin Konijnenbelt, Henry Mulder, Cees M P M Hertogh, Maurits van Tulder, Gert Kwakkel, Erwin van Wegen

**Affiliations:** Amsterdam Rehabilitation Research Centre | Reade, Amsterdam, The Netherlands; Department of Rehabilitation Medicine, MOVE Research Institute Amsterdam, VU University Medical Center, Amsterdam, The Netherlands; AptaVivar, Hengelo (Ov.), The Netherlands; Department of General Practice and Elderly Care Medicine and the EMGO Institute for Health and Care Research, VU University Medical Center, Amsterdam, The Netherlands; Department of Health Sciences & EMGO+ Institute for Health and Care Research, Faculty of Earth & Life Sciences, VU University, Amsterdam, The Netherlands

**Keywords:** Stroke, Caregiver, Exercises, Early supported discharge, Mobility, e-health, Rehabilitation, Cost-effectiveness

## Abstract

**Background:**

Several systematic reviews have shown that additional exercise therapy has a positive effect on functional outcome after stroke. However, there is an urgent need for resource-efficient methods to augment rehabilitation services without increasing health care costs. Asking informal caregivers to do exercises with their loved ones, combined with e-health services may be a cost-effective method to promote early supported discharge with increased functional outcome.

The primary aim of the CARE4STROKE study is to evaluate the effects and cost-effectiveness of a caregiver-mediated exercises program combined with e-health services after stroke in terms of self-reported mobility and length of stay.

**Methods:**

An observer-blinded randomized controlled trial, in which 66 stroke-patients admitted to a hospital stroke unit, rehabilitation center or nursing home are randomly assigned to either 8 weeks of the CARE4STROKE program in addition to usual care (*i.e.*, experimental group) or 8 weeks of usual care alone (*i.e.*, control group). The CARE4STROKE program is compiled in consultation with a trained physical therapist. A tablet computer is used to present video-based exercises for gait and gait-related activities in which a caregiver acts as an exercise coach.

Primary outcomes are the mobility domain of the Stroke Impact Scale and length of stay. Secondary outcomes are the other domains of the Stroke Impact Scale, motor impairment, strength, walking ability, balance, mobility, (Extended) Activities of Daily Living, psychosocial functioning, self-efficacy, fatigue, health-related quality of life of the patient as well as the experienced strain, psychosocial functioning and quality of life of the caregiver. An economic evaluation will be conducted from the societal and health care perspective.

**Discussion:**

The main aspects of the CARE4STROKE program are 1) increasing intensity of training by doing exercises with a caregiver in addition to usual care and 2) e-health support. We hypothesize this program leads to better functional outcome and early supported discharge, resulting in reduced costs.

**Trial registration:**

The study is registered in the Dutch trial register as NTR4300, registered 2 December 2013.

## Background

Stroke poses major social and healthcare problems worldwide. The prevalence of stroke is increasing [1]. In 2010, the absolute numbers worldwide of people with first stroke (169 million), stroke survivors (33 million), stroke-related deaths (59 million), and DALYs lost (102 million) were high and had significantly increased since 1990. About 28 % of stroke patients remain dependent in basic activities of daily living (ADL) such as dressing, toileting and/or indoor mobility at twelve months after stroke [2]. Although the main target of stroke rehabilitation is to reduce long term dependency and allow patients to return to their own community [3], only 60 % of the stroke patients can ultimately walk independently with or without assistive devices in the community [4].

In the 27 EU countries, total annual cost of stroke is estimated at €27 billion: €18.5 billion (68.5 %) for direct and €8.5 billion (31.5 %) for indirect costs. A further sum of €11.1 billion is calculated for the value of informal care [5]. The already overstretched health resources worldwide emphasize the need for early supported discharge (ESD) of stroke patients [3, 6], because a large part of the stroke care costs are spent on inpatient rehabilitation services [7, 8].

A large number of stroke patients are using inpatient services because they are not safe and independent in their mobility. ESD is enabled as soon as these patients are safe and independent in their transfers and gait, suggesting that ESD heavily depends on improvement of standing balance and motor control of the lower limbs [3, 9].

A number of meta-analyses show that intensity of training and repetitive task training are crucial aspects of stroke rehabilitation, concluding that more exercise therapy improves outcomes [3, 10–16]. Guidelines recommend that patients admitted to a rehabilitation facility should have the opportunity to receive a daily dose of 45 min of exercise therapy in the first 3 months after stroke [15, 17–20]. However, most patients admitted to hospital stroke units, rehabilitation centers or nursing homes are physically inactive or involved in activities that contribute little to their recovery [21–23]. A recent survey in the Netherlands of 91 hospital stroke units showed that patients receive on average about 24 min of exercise therapy each working day [24].

Acknowledging that the resources (mostly staff) in rehabilitation settings are limited, novel methods to increase the intensity of exercise therapy with minimal use of resources are needed [3, 24]. One such novel method could be to actively involve caregivers in mediating exercises. In particular when caregiver-mediated exercises (CME) are combined with e-health/tele-rehabilitation services, easy contact with, and monitoring by the rehabilitation team is promoted [25]. This way, CME enhances ESD by providing a smoother transition from inpatient setting to the home situation. And CME can continue in the community setting.

Recently, Galvin *et al.* found favorable effects of CME on functional outcome in stroke patients and on perceived strain by caregivers [26]. In addition, we hypothesize, CME might contribute to improved feelings of quality of life (QOL) and empowerment for both patient and caregiver by providing them with more knowledge about the capabilities of the stroke patient.

Few randomized controlled trials (RCTs) have investigated CME and their quality is heterogeneous [26–31]. In addition, CME has not been combined with e-health facilities to promote self-management and empowerment of patient and caregiver, whereas studies investigating cost-effectiveness of CME after stroke are still lacking.

The aim of the current paper is to describe the CARE4STROKE study design. The CARE4STROKE study aims to evaluate the effects and cost-effectiveness of a CME program combined with e-health, added to usual care in hospital stroke units, rehabilitation centers and nursing homes. We hypothesize that the CARE4STROKE program will lead to better self-reported mobility and reduced length of inpatient stay (LOS) in stroke patients compared to usual care, resulting in reduced costs.

## Methods

### Design

This study is an observer-blinded, multicenter randomized controlled trial with an economic evaluation alongside. The trial will be conducted by trained therapists of the participating centers. Within each type of setting, patients will be randomly allocated to either CME combined with e-health services (CARE4STROKE) in addition to usual care or to usual care alone. The study is registered in the Dutch trial register as NTR4300, registered 2 December 2013.

### Setting

The study will take place in hospitals (stroke unit and outpatient clinic), rehabilitation centers and rehabilitation departments of nursing homes in the Netherlands.

A trained researcher, blinded to group allocation, will visit the participants in the center of admission for obtaining informed consent and conducting measurements during the study.

Reade Rehabilitation Center and VU University Medical Center are the initiators of this study. The study protocol is approved by the Medical Ethics Review Committee of the Slotervaart Hospital and Reade and is registered with the trial number: NL34618.048.12.

### Participants

Sixty-six patients with a first-ever or recurrent stroke, who are admitted to one of the participating centers and their caregivers, will be recruited for this study. Stroke is defined by the World Health Organization as "a clinical syndrome typified by rapidly developing signs of focal or global disturbance of cerebral functions, lasting more than 24 h or leading to death, with no apparent causes other than of vascular origin" [32]. A caregiver is defined as someone close to the patient, who is willing and able to do exercises together with the patient, for example a partner, family member or friend. This caregiver is not a professional and is not paid for his/her efforts.

Inclusion criteria for both patient and caregiver are: (1) 18 years or older, (2) written informed consent, (3) able to understand the Dutch or English language (on a sufficient level to understand instructions on CME and e-health application), (4) motivated for CME, (5) a score of <11 on the domain ‘depression’ on the Hospital Anxiety and Depression Scale (HADS) [33, 34].

Additional inclusion criteria for the patient are: (1) willing and able to appoint a caregiver who wants to participate in the program (with a maximum of two caregivers), (2) living independently before the stroke, (3) planned to be discharged home, (4) being able to follow instructions (MMSE score > 18 points) [35], (5) Functional Ambulation Score (FAC) < 5 [36].

Additional inclusion criteria for the caregiver are: 1) being medically stable and 2) physically able to perform the exercises together with the patient.

Exclusion criterion for both patient and caregiver will be serious comorbidity, which interferes with mobility training. Patients will be excluded when they, for example, have another neurological disease like Multiple Sclerosis or Parkinson disease, fractures or congestive heart failure. Caregivers will be excluded when they are not able to walk 100 metres, stand and/or keep their balance.

To determine suitability in terms of safety, cognition and communication skills of both patient and partner, an intake exercise session with one of the trained physical therapists is scheduled prior to inclusion. This therapist checks the inclusion/exclusion criteria and judges if the exercises can be done adequately and safely. Reasons of exclusion will be recorded.

#### Baseline characteristics

Patient characteristics at baseline will be recorded from the medical status. These include demographics (age, gender), type of stroke, time post stroke, hemiplegic side, somato-sensory deficits (yes/no), homonymous hemianopia (yes/no), visuo-spatial neglect (yes/no), aphasia (yes/no) and comorbidity following the Cumulative Illness Rating Scale (CIRS) [37]. Characteristics of the caregiver that will be recorded are: demographics (age, gender), relation to patient, work and existing comorbidities following the CIRS.

### Study procedures

**Fig. 1:**
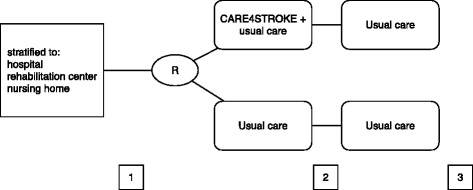
Study design. R = Randomization. 1 = Measurement 1, baseline, before start of the intervention. 2 = Measurement 2, end of intervention (eight weeks post randomization). 3 = Measurement 3, follow up (twelve weeks post randomization)

Patients will be stratified by type of participating center (hospital stroke unit, rehabilitation center or nursing home) and subsequently randomized, by an independent researcher blinded for patient characteristics, to the control or the experimental group. An online randomization procedure with a minimization algorithm is used to prevent unequal group sizes [38]. Patients will start immediately after admission, continue for 8 weeks irrespective of time of discharge and will be followed-up until 12 weeks after randomization. Outcomes will be measured at baseline, 8 and 12 weeks (see Fig. 1: Study design). Outcomes are either self-reported by patients and caregivers (not blinded) or measured by an independent observer who is blinded for treatment allocation. A self-reported (cost) diary will be kept during the intervention period to monitor compliance and to collect relevant cost-data.

### CARE4STROKE intervention

The CARE4STROKE program consists of 8 weeks of exercise therapy executed with a caregiver, in addition to usual care. A total of 37 standardized exercises were developed that are aimed at improving mobility skills related to walking like, standing, turning and making transfers, or are supporting exercises to improve mobility, strength and (sitting) balance. Subsequently, exercises can be combined into a patient-tailored, weekly progressive and incremental training regimen. All exercises were developed in collaboration with rehabilitation specialists (movement scientists, physical therapists and physicians) and have been shown feasible in a pilot study.

The exercises are presented as instructional videos in an e-health application (‘app’) on a tablet computer. All exercises are explained by a voice over. Regular reminders to exercise can be set in the app. The patients and their caregivers are asked to perform the selected set of exercises minimally five times per whole week for 30 min. Patients and caregivers are in particular advised to do the exercises during the weekends, acknowledging that patients are often physically inactive during the weekend. When the intervention is correctly performed patients will thus have a surplus of 150 min of caregiver-mediated exercise training during a whole week.

During the intervention period patients and their caregivers will have a weekly session with one of the trained physical therapists. In these sessions, the set of exercises performed in the previous week is evaluated and adapted in a progressive manner. The participating couple is subsequently instructed as to which set of exercises should be performed during the next week. To make sure exercises are correctly performed, the therapist will give instructions about the new exercises and the patient-caregiver couple will be asked to do these exercises in the presence of the therapist during this session. The therapist will register all planned exercises and also if any adverse event happened during the last week. Furthermore, patient - caregiver couples are encouraged to contact the coordinating therapist through telephone, skype or email when appropriate.

The CARE4STROKE program starts when the patient is admitted to one of the participating centers. When the discharge date of the patient is earlier than the anticipated end date of the CME program, the CME program continues at home with the continuity of the use of the app, the weekly sessions with the therapist and the possibility to contact the therapist through tele-rehabilitation services when appropriate.

#### Usual care

The participants in the control group will receive usual care according to the Guidelines of Physical Therapy for patients with stroke of the Royal Dutch Physical Therapy Association KNFG [15].

#### Compliance

In order to conduct this trial uniformly in the different centers, all participating therapists will be thoroughly trained in a training course before they start delivering the program. Each therapist will be informed about 1) the aims, design and measurements of the CARE4STROKE study, 2) the in- and exclusion criteria, 3) the CARE4STROKE program: the standardized program and the possibilities to customize the CME, 4) their role in the intake exercise session and following exercise sessions, 5) how to fill in the diaries, 6) the use of the app. Regular retraining sessions will be organized for the participating therapists. A researcher (RN) will monitor if the intervention is implemented appropriately in the participating centers.

A self-reported diary will measure compliance of patient and caregiver with the CME program.

### Outcome measures

Primary outcome measures are the mobility domain of the Stroke Impact Scale (SIS 3.0) and LOS.

#### Stroke Impact Scale (SIS) version 3.0, Mobility domain

The SIS is a self-reported, stroke specific measure that includes 59 items and assesses eight domains related to activities and participation. The mobility domain of the SIS includes questions about patients’ perceived competence to walk, keep balance, and move around in their own community. Each item is scored from ‘not difficult at all’ to ‘cannot do at all’ on a 5-point scale. The SIS has shown excellent clinimetric properties in English, as well as in the Dutch translation [39–43].

#### Length of Stay (LOS)

LOS will be defined as the number of days of inpatient stay in a rehabilitation facility and/or hospital setting, from the day of admittance until the day of discharge. Mean length of stay for each setting will be determined. Possible reasons for an extended inpatient stay, like medical complications or time needed for the realisation of facilities at home, will be recorded.

### Secondary outcomes

#### * Patient

##### Stroke Impact Scale, other seven domains

The other self-reported domains of the SIS will be assessed as secondary outcome measures.

##### Fugl Meyer (FM) motor score of lower extremity

The FM will be used to assess motor impairment. It is a reliable and valid motor performance test and evaluates the ability to make movements outside the synergistic movement pattern [44].

##### Motricity Index (MI), lower extremity

The MI is a valid and reliable measure of the strength of the lower extremity. Scores range from 0 (no activity) to 33 (maximum muscle force) for each dimension [45].

##### Six minute walking test

Gait performance and endurance will be assessed by the six minute walking test [46, 47]. The walking distance covered in six minutes will be recorded.

##### Ten meter walking test

Gait speed will be measured by the ten meter walking test. Comfortable walking speed will be assessed. The mean of three repeated walking speed measurements will be calculated [46, 47].

##### Timed up and go test (TUG)

The TUG is a test of basic functional mobility. The participant is asked to rise from an armchair, walk three metres as fast as possible, cross a line, turn, walk back and sit down again. The time to complete this test will be recorded [46, 47].

##### Berg Balance Scale (BBS)

Balance will be evaluated by the BBS. BBS is a widely used clinical test of a person's static and dynamic abilities. There are 14 items scored from 0–4 (maximum), with a total score of 56. The BBS is a valid and reliable measure [48, 49].

##### Rivermead Mobility Index (RMI)

The RMI is a test to evaluate functional mobility. It consists of 14 questions and one observation (of balance) covering aspects from turning in bed to running. The questions are scored dichotomously. The RMI is valid, reliable and responsive [50–52].

##### Barthel Index (BI)

The BI is an ordinal scale to measure performance in activities of daily living (ADL). It uses ten variables describing ADL and mobility. A higher score indicates higher independence in ADL. It has excellent clinimetric properties and can also be filled out by an experienced nurse or relative [53].

##### Nottingham Extended ADL scale (NEADL)

The NEADL is a self reported questionnaire on activities actually performed. It consists of 22 items in four domains (mobility, kitchen, domestic, leisure). Each item is rated by one of four responses (able, able with difficulty, able with help, unable). The NEADL has proved to be a reliable and valid outcome measure in patients with stroke [54].

##### Modified Rankin Scale (MRS)

The MRS is a measure for the degree of disability or dependence in the daily activities. The score runs from 0–6, ranging from perfect health without symptoms to death. The score will be dichotomised to good outcome (0–2) or poor outcome (3–6). It is a valid scale and frequently used in stroke outcome studies [55].

##### EuroQol (EQ-5D)

The EQ-5D measures health related quality of life. It consists of a self-assessment questionnaire about current health in five dimensions (mobility, self-care, usual activities, pain/discomfort and anxiety/ depression) and a VAS score in which a person is asked to rate their own health status. It is a widely used generic questionnaire, which is validated for people with stroke. By combining the questionnaire and VAS score a health state is described, and each health state combined with population estimates can be transformed to a utility. A utility is an expression of the Quality Adjusted Life Years (QALY) and can be used in economic evaluations [56–59].

#### * Caregiver

##### Expanded Caregiver Strain Index (CSI+)

The CSI+ evaluates experienced strain of the caregiver. There are 18 items answered with ‘yes’ or ‘no’ and scored dichotomously. The CSI+ is an expansion of the Caregiver Strain Index and also rates positive aspects of caring. The CSI+ is proven valid and responsive [60–62].

##### Carer Quality of Life Scale (CarerQOL)

The CarerQOL is a valid instrument to evaluate care-related quality of life in informal caregivers. The instrument consists of a burden instrument (encompassing seven important burden dimensions) and a valuation component (a VAS scale for happiness). It consists of seven questions with each three-answer options (no, some, a lot) and a VAS scale (‘how happy are you at this moment?’) [63–65].

#### * Patient and caregiver

##### Hospital Anxiety and Depression Scale (HADS)

The HADS is a measure to evaluate mood: anxiety and depression. The HADS consists of 14 items (seven anxiety and seven depression), each with a 4-point rating scale (0–3) It is a brief, reliable, responsive, valid and widely used measure [33, 34].

##### Fatigue Severity Scale (FSS)

The FSS measures the impact of fatigue. It consists of nine items, and scores for each item range from 1 to 7. The total FSS score is the mean of the nine item scores. The FSS was validated and demonstrated to be a simple and reliable instrument to assess and quantify fatigue for clinical and research purposes [66].

##### General Self-efficacy Scale

The General Self-Efficacy Scale is a valid 10-item psychometric scale that is designed to assess optimistic self-beliefs to cope with a variety of difficult demands in life. It has a 4-point rating scale (‘not at all true’. ‘barely true’, ‘moderately true’ and ‘exactly true’). The scale has been originally developed in German and has been used in many studies with hundred thousands of participants. General self-efficacy is a universal construct that yields meaningful relations with other psychological constructs [67, 68].

##### (Cost) Diaries

Each patient-caregiver couple will be asked to keep a weekly cost diary during 12 weeks. Direct and indirect cost data will be collected. The diary will comprise questions for the patient on medical consumption (for example questions about consultation with doctors, therapists, re-admission, home care), missing hours at work, household, sports or hobbies and time invested by the caregiver in the caregiver-mediated training [69]. In addition, the patient will be asked to record the exercises done each day during the eight-week intervention period (in therapy, by themselves, with nurses or with a caregiver). Thereby we can evaluate the total time spent on (additional) exercises done by the couples in the intervention and control group. Problems and adverse events like for example falls, fractures, and concurrent illness will also be recorded.

### Process analysis

At the end of the intervention, semi-structured interviews will be conducted with a subgroup of patients and caregivers to collect qualitative data regarding the experience of CME to evaluate facilitators and barriers for implementation.

### Power analysis

We expect a significant reduction of five points (11 %) on the SIS mobility domain in favor of the experimental training group, with an estimated standard deviation for this population at a maximum of 14 points [70], requiring inclusion of minimally 30 patients per arm of the trial. Including 10 % dropouts, a minimum of 66 stroke patients, (*i.e.,* 22 per type of center), is needed to achieve a sufficient statistical power of 80 % using a significance level alpha of p < 0.05.

### Data analyses

Baseline characteristics will be presented and between group differences will be studied to determine whether groups are comparable at baseline. Normality of data distributions will be judged by visual plot. When data are not normally distributed, non-parametric Wilcoxon signed rank sum tests will be used. When the data are normally distributed student t-tests for independent samples will be used. The two-tailed α-level will be set at 0.05.

The main outcomes will be compared between the intervention and control group at the different time points using multilevel regression analysis. Depending on the number of settings of participating centers we will use random coefficient analysis (SPSS GLM). Time since stroke, group, location and baseline values will be added to the model. Intention-to-treat analysis will be done and missing data will be imputed using multiple imputation techniques. All hypotheses will be tested two sided, with a critical value of <0.05.

### Economic evaluation

The economic evaluation will be performed from a societal perspective and a health care perspective with a time horizon of 12 weeks. For the measurement and valuation of the costs the Dutch costing guidelines will be used [71]. All relevant costs will be measured and valued, including cost of production loss where applicable. The analysis will be done according to the intention-to-treat principle. Missing cost and effect data will be imputed using multiple imputations according to the MICE (Multiple Imputation by Chained Equations) algorithm [72]. Bias-corrected and accelerated bootstrapping with 5000 replications will be used to calculate 95 % confidence intervals around the mean difference in total costs between the two groups. Incremental cost-effectiveness ratios (ICERs) will be calculated by dividing the difference in mean total costs by the difference in mean effects on the primary outcomes (SIS mobility and LOS) between the treatment groups. A cost-utility analysis will be performed estimating the incremental costs per QALYs gained. In the costs utility-analysis the outcome measure will be QALYs based on the Dutch tariff for the EuroQol [56]. Bootstrapping will be used to estimate the uncertainty surrounding the ICERs, which will be graphically presented on cost-effectiveness planes. Cost-effectiveness acceptability curves and net monetary benefits will also be calculated. To estimate indirect costs of production loss the human capital approach will be used. Sensitivity analyses will be performed on the most important and uncertain cost parameters.

## Discussion

The CARE4STROKE trial is the first observer-blinded randomized clinical trial aimed to investigate the effectiveness of a CME program combined with e-health, added to usual care, in terms of self-reported outcome of mobility (SIS 3.0) and LOS in patients with stroke admitted to hospital stroke unit, rehabilitation center or nursing home.

The main aspects of the CARE4STROKE program are 1) increasing intensity of training by doing exercises with a caregiver in addition to usual care and 2) e-health support.

A higher intensity of training improves functional outcome after stroke [3, 10–16]. However only few studies have been done in which a higher intensity of training is achieved by CME [26, 29–31]. Moreover, none of these existing studies investigated the cost-benefits of CME and none combined CME with e-Health services. We assume that e-health can support adherence to the program for patient and caregiver and promote self-management [25, 73]. In this intervention e-health consists of the CARE4STROKE tablet application, which clearly explains the exercises through video instructions and is simple and attractive to use. And, in addition to this, tele-rehabilitation services are available such as telephone, skype or email to contact the coordinating therapist when appropriate.

We hypothesize that the combination of a weekly progressive, incremental training regimen done with a caregiver together with continuing support of a therapist through additional e-health services may enhance ESD and increase feelings of QoL, perceived empowerment and self-management of the patient - caregiver couple. As a consequence, we expect that CARE4STROKE will lead to a reduced LOS and will thereby reduce care costs.

Defining LOS should be done carefully, since it may be influenced by non-medical factors that are not directly related to the functional ability of the patient. Data on additional factors which could also influence LOS will be collected, like discharge destination, comorbidity, the need for facilities at home, *etc.* [74]. We will also record the planned discharge date and the real discharge date. We will describe these data and, when necessary, do subgroup analyses.

The optimal dose for caregiver-mediated exercises is not yet known, few studies have been done [26–31]. We ask patients and caregivers to perform the selected set of exercises minimally five times a week for 30 min. This dose was chosen because it leads to a surplus of 150 min of exercise a week, which is in line with recommendations of most guidelines [15, 17–20] and proved to be feasible in our pilot study.

Caregivers are more intensively involved in CME than during usual care. At first glance, this could increase caregiver strain. Other studies show no significant negative influence or even a decrease in caregiver strain in the CME intervention group [26, 30]. It is suggested that the latter effect arises due to more knowledge and experience of the caregiver about what the patient can and cannot do. We will not only assess caregiver strain, but also anxiety, depression, quality of life, fatigue and self-efficacy of the caregiver, to closely monitor the effects of our intervention on the caregiver.

CME will be implemented in three different rehabilitation settings, *i.e.,* hospital stroke units, rehabilitation centers and nursing homes, to study its applicability and effectiveness in different care settings. The inclusion criteria are liberally defined, in order to get more insight in the type of patients and caregivers that are eligible for CME and facilitators and barriers for implementation. For example we will include patients with MMSE > 18 [35] and patients and caregivers with a HADS score on the domain ‘depression’ < 11 [33, 34], acknowledging that patients with some cognitive decline and patients and caregivers with some depression may benefit from our CARE4STROKE program. Probably a major factor for successful implementation is that the patient and caregiver are physically and emotionally able and willing to perform the exercises together. Therefore, an intake exercise session with the physical therapist is incorporated to judge whether patient and caregiver can adequately perform the exercises together.

Interestingly, in a small-scale pilot study, about 25 % of the eligible participants with stroke, did not have a willing and/or able caregiver. We will keep track of inclusion-rates and reasons for exclusion and will record how many possible participants cannot continue because of a lack of caregivers. This is relevant data in light of a trend in transferring care from professional to informal caregivers with focus on self-management and has not been addressed in previous RCTs on CME [26–31].

A limitation of our current design is that participants in the control group continue with usual care but do not receive any (new) intervention. With that the trial is not dose- matched. In addition, the adherence of the participants included in the control group to fill in the diaries could be low. Furthermore, in some centers, participants in control and intervention groups are admitted to the same wards. This could lead to contamination in the control group, when patients and/or caregivers see how others exercise together. Finally, our selection criteria are liberally defined, this could be a limitation of external validity. During the training course, participating therapists are instructed to pay specific attention to the diaries and dangers for contamination to minimize these effects. A number of outcome measures, including one of the primary outcome measures (mobility domain of SIS 3.0), are self-reported. It is impossible to blind the patient-caregiver couple, and therefore these outcome measures are not blinded. However, a blinded outcome assessor assesses the other objective outcome measures.

In conclusion: The CARE4STROKE study will be the first clinical trial in which the effects and cost-effectiveness of CME combined with e-health services to enhance ESD is investigated. The first results are expected early 2018.

## Abbreviations

ADL: Activities of Daily Living
BBS: Berg Balance Scale
BI: Barthel Index
CME: Caregiver-Mediated Exercises
CSI+: (Expanded) Caregiver Strain Index
ESD: Early Supported Discharge
FAC: Functional Ambulation Categories
FM: Fugl Meyer
FSS: Fatigue Severity Scale
HADS: Hospital Anxiety and Depression Scale
ICER: Incremental cost-effectiveness ratio
KNGF: Koninklijk Nederlands Genootschap voor Fysiotherapie
LOS: Length of Stay
MI: Motricity Index
MICE: Multiple Imputation by Chained Equations
MMSE: Mini Mental State Evaluation
MRS: Modified Rankin Scale
NEADL: Nottingham Extended ADL Index
QALY: Quality Adjusted Life Year
QOL: Quality of Life
RCT: Randomized Controlled Trial
RMI: Rivermead Mobility Index
SIS: Stroke Impact Scale
TUG: Timed Up and Go Test
